# Monthly Summary

**Published:** 1900-04

**Authors:** 


### Monthly Summary.

MICROBES IN THE ARCTIC REGIONS.—Recent explorers in both the Arctic and Antarctic regions have brought back interesting information concerning bacterial life in the zones. It has long been known that travelers in the Arctic territory suffer very little from excessive changes of temperature, and are entirely free from colds and coughs which are so frequently observed in the winter in our own latitude. Nordenskiold is authority for the statement that Spitzbergen in the summer time is the healthiest portion of the earth. Levin made a number of cultures of the air in Spitzbergen and in King Charlesland. Samples of air were taken on the surface of the glacier, on the coast, on the top of a cliff, as well as on board ship. In each instance at least 1,800 liters (nearly 50 gallons) of air was filtered, indicating an elapsed time during the experiment of four or five hours. In only one instance were bacilli found. In that case the air was taken from the deck of the vessel while it was in harbor, and as only three colonies of bacteria developed, it is at least a question whether a grain of dust from the ship did not get into the gelatin. On the other hand, all samples of water, whether taken from the surface of the sea or at a great depth, or from a glacier or obtained by melting snow or ice, were found to contain bacteria, although in very small numbers. At the surface of the sea Levin found one germ for each 11 c.c. (3 drams) of water—a quantity of germ-life which is absolutely insignificant. The same amount of water taken from the River Seine has been found to contain more than two million bacteria. A curious fact was noticed in that the water taken from the ocean at great depth invariably contained more bacteria than water from the surface, and this in spite of the fact that deep water in the Arctic Ocean is usually below the freezing point. Levin made another series of experiments in order to determine the bacterial condition of the intestinal contents of various animals, white bears, seals, reindeer, elder ducks, penguins, gulls, frigate birds, sea-urchins, sea-anemones, shrimps; etc. These experiments showed him that in most of these animals the contents of the intestines are almost entirely sterile. In one white bear and in two seals was found a species of bacteria which resembled the bacillus coli commune. The inferior animals, sea-urchins, sea-anemones, etc., usually contained bacteria.

While scientists have long held that bacteria are not indispensable to digestion it is extremely interesting to receive this proof of their statement direct from the natural world. This fact and the fact of the existence of a whole world of bacterial life at a temperature sometimes 3½° F. below the freezing point are the most valuable results of Levin's researches, a full report of which will be found in the July number of the "Annales de l' Institut Pasteur."—"Dental Brief."

---

THE STERILIZATION OF WATER BY MEANS OF OZONE has now been in operation at Lille for some months, and a Commission, including Drs. Roux and Calmette, has recently issued a report on its efficiency. This ozone was produced electrically and was thoroughly mixed wlth the water, the amount present varying from 5 to 10 mgms. of ozone per litre of air. The amount of water treated was 35 cubic meters per hour. The conclusions of the commission are very favorable. The apparatus worked well and simple, and brought about a marked sterilization of the water. The untreated water contained over 2,000 organisms per cubic centimeter, after passing through the ozonier it cantained only two or three organisms (B. subtilis) per cubic centimeter.—"Pharmaceutical Journal."

---

A NEW ALLOY is being used in France in the construction of the bogies of automobile vehicles. It has been used by De Dion and Bouton, and has been given the name of Partinium. It is an alloy of aluminum with tungsten, and gives a metal with a specific gravity of 2.89 when cast and 3.09 when rolled. It is said to be stronger than aluminum, and almost as light, while at the same time it is less expensive.—"Dental Brief."

PLAIN TEETH WITH PINK RUBBER.—Objection is sometimes urged to the liability which the red or black vulcanite of which the plate is made, cropping through on the pink rubber gums and marring the effect. This would certainly be an objection if it could not be successfully combatted. The cropping through of red or black rubber on the pink gum facing may always be avoided, if the case be first carefully packed with pink rubber, especially between the teeth, as they lie in the plaster investment of the flask, using a thin instrument to pack the pink rubber (which should be softened on a heated soapstone slab) in these spaces. After the case is thus packed—packing the pink rubber of the gum facing first—the red or black rubber is packed afterwards, gates for the escape of the surplus rubber having been cut entirely the back of the investment, no gates whatever being cut on the front. The two parts of the flask are now put together and the bolts applied, and the flask immersed in water and permitted to boil at least five minutes. The flask is then lifted out of the boiling water, and the two front bolts screwed down first. When these are down as far as they will go, the flask is boiled again and the bolt screwed down. If a case of plain teeth with pink rubber gums be manipulated in this way the red or black rubber of the plate will never crop through to deface the gum facing.—T. F. Chupein, in Office and Laboratory.

---

THE USEFUL LEMON.—The relations of fruit to digestion are particularly interesting. Fruit juices are disinfectants. They are germicidal. The juice of the lemon is as deadly to cholera germs as corrosive sublimate, or sulphur fumes, or any other disinfectant. It is so powerful a germicide that if the juice of one lemon be squeezed into a glass of water that is then left standing ten or fifteen minutes, the water will be disinfected; it makes little difference where the water has been obtained, or whether it has been boiled or filtered. This is a fact worth knowing, for any one of us may find himself under circumstances in which it is impossible to get either boiled or filtered water. In such a case, the juice of a lemon will purify the water perfectly.—Good Health.

SENSITIVE DENTINE.—Sensitive dentine is not so difficult of control as some suppose. A little paste of oil of cloves, creosote and tannin on cotton, with a few finely crushed crystals of cocaine on the surface of the paste is good. Let it remain in the cavity two or three days covered with cotton moistened with sandarach varnish.

For immediate and temporary effect let the patient press on the tooth a little oil cloth or rubber bag filled with pulverized ice—first slightly, and gradually more closely until the tooth is benumed.

But for obstinate, deep-seated, sensitive decay, and for a permanent cure, where the pulp is not exposed or nearly so, there is perhaps nothing better than a few fine scrapings of nitrate of silver, substituted for the cocaine in the above treatment. It will not only remove the sensitiveness, but will permanently harden the dentine, so as to prevent further decay. I have in this way removed sensitiveness of superficial decay on the surface and neck of teeth and afterward found the affected part so hard as to need but little further treatment, and no filling.—Dental Brief.

---

DENTISTRY IN FRANCE.—This sign—"Dr. Sylvester, American Dentist"—at the entrance to his office in a French city resulted in the condemnation of the dentist on two indictments: 1, for practicing under a pseudonym, as his name was in reality Sylvester Baumgartner; and 2, for neglecting to append the source of his medical diploma, the court asserting that dentistry being a branch of medicine the derivation of the title of "Dr." must be stated on the sign to conform to the French law in respect to aliens practicing in France.—"Dental Brief."

---

CHLOROFORM IN INDIA.—It has been found that an apparatus for killing animals with chloroform in England would not work in India, because the high temperature prevented the concentration of the chloroform vapor. That this was the case was proved by the fact that by placing ice in the box the animals were readily killed.—"Dental Brief."

'DENTISTRY" VS. "MECHANICAL DENTISTRY."—Whether it is done intentionally or not, there are licentiates who seem determined to drag down prosthetic dentistry to the level of a trade. Trade methods, and the catch-penny advertisements of the traders, are introduced into the press, and by vulgar showcases, just as we are accustomed to seeing with the display of boots and shoes, the trade ideas are emphasized. The veterinary surgeon needs the environment of the stable. Some of them go so far as to advertise themselves as blacksmiths in addition. These are legitimate and honorable, but veterinary surgery, *per se*, is a scientific profession, which any educated gentleman might be ambitious to practice. It may come to pass that mechanical dentistry will be assigned the same relationship that the forge occupies to some of the Vets. There will always be those, however, who will then have only a collateral interest in prosthetic dentistry proper, and as the oculist sends his "prescription" to the optician, and the surgeon his legless and armless patients to the manufacturer of artificial limbs, so the distinctively "surgeon" dentist may yet relegate his prosthetic cases to the exclusive mechanic. Long ago we foreshadowed this, and it looks now as if it were one of the changes sure to come in the course of time.—"Dominion Dental Journal."

---

A YOUNG licentiate of Quebec had practiced for one year in a small village of considerable wealth, yet of rather narrow intelligence regarding the value of conservative dentistry, Fully three-fourths of his patients believe that the fakir who perambulated the country, crying up his cheap artificial sets of teeth, and crying down all efforts to save the natural teeth, was a missionary of mighty peace and enduring comfort. Not a day passed that the conservative practitioner did not meet this class, and business left him because he would not extract teeth wholesale. At the end of the year he decided to leave the unsavory pastures and find a better field. One of the resident clergymen came to see me. "I'm told, doctor, that you are going to leave us ? May I ask the reason ?" "Yes," replied the dentist. For the first six months

I told the truth, the whole truth, and nothing but the truth, to my patients, and I nearly starved. For the last six months I have told them anything but the truth. I've trebled my income, but I'm nearly choked. The dentist who can make lying a profession is the sort of a man to succeed here." And he left.—*Dominion Dental Journal.*

---

SKULLS AND BRAIN CAPACITY.—Professor Arthur Thompson, in the October number of "Knowledge", deals with the form of skulls and brain capacity. The average weight of a man's brain is about fifty ounces; that of a woman about forty-five ounces. This difference between the sexes is ess marked in savage than in civilized races, and is apparently explained by the fact that in the higher races more attention is paid to the education of the male than the female, and consequently the brain is stimulated to increased growth.—"Dental Brief."

---

REPAIRING RUBBER PLATES.—Allow me a little space to describe a simple method of repairing rubber plates. With wax fasten the fractured pieces together and insert the palatal surface in plaster. Cut a narrow section through the fracture and burrow the adjoining surfaces one-eighth inch deep and one-quarter inch wide. No holes, dovetails, nor grooves are needed, but simply pack the fresh rubber with a hot spatula and flask.—L. P. Haskell in "Dental Brief."

---

REAL diamonds cost more than paste diamonds. You must pay more for silk than for cotton. The jeweler who says he gives you real diamonds at the cost of paste, the dry goods merchant who says he gives you silk at the price of cotton, are just like the dentist who says he will give you the best sets of teeth at the price of the poorest.—"Dominion Dental Journal."

STERILIZING DENTAL INSTRUMENTS.—While boiling, even when disinfectants are used, cannot be relied on for destroying all forms of germ life, on the other hand, it has been conclusively demonstrated that steam without the use of disinfectants will most effectively destroy organisms which cause infectious disease. For steaming dental instruments and napkins, thereby more thoroughly sterilizing than by simply boiling them, the ordinary dental vulcanizer furnishes a simple and effective sterilizer.

Place the instruments in cotton or linen bag tied closely at the top, put in the vulcanizer, and run the thermometer up to 230°. Shut off the gas, and allow the instruments to remain in the steam bath for ten minutes. A small quantity of bicarbonate of soda placed in the water will prevent the instruments from rusting. Any vulcanizer holding three flasks will admit the forceps, which of all dental instruments should be thoroughly sterilized.—Kasson C. Gibson, in *Cosmos*.

---

ANCHYLOSIS OF THE JAW DUE TO INTERSTITIAL MYOSITIS.—Seggel (*Duetsch. Zeit. f. Chir.*, May, 1899) describes a case of anchylosis of the jaw of rather unusual character. The patient was a woman aged forty-eight years, who lost all of the molar teeth on the left slde on account of caries. They were pulled out in a rough manner and marked inflammation followed each extraction. Three years after their removal the patient was exposed to a severe wetting and was thoroughly chilled. The left side of the face swelled badly, and this swelling and pain lasted for more than three months. From that time on the cheek never regained its normal size, but grew gradually larger without pain until it was impossible to open the jaw. A diagnosis of sarcoma of the ascending portion of the jaw was made, and an extensive resection or the bone and soft parts, including the whole of the parotid gland, was carried out. Complete facial paralysis of that side resulted. The wound healed well, and in six weeks the patient could open her mouth nearly an inch. The tendency of the jaw to fall to the right side was counteracted by a plate with an inclined surface. Microscopical examination of the excised muscle showed that its fibers were separated by a large amount of cicatricial tissue and the fibers themselves were atrophied. The question of syphilis, after careful consideration, was rejected. The lesions appeared to be due to traumatism at the time of the extraction of the teeth and to the inflammation caused at that time, and after the exposure to the weather. Several similar cases have been reported, although usually anchylosis of the jaw is due to syphilis, or to myositis ossificans progressiva, or rheumatism.—*Medical News*.

---

LEGRAND'S SOLUTION ANESTHESIQUE-HEMOSTATIQUE has the following formula :

| | | |
|---|---|---|
| ℞ | Gelatin. pur. - - - | 2.0 |
| | Sodii chloridi. - - - | 0.7 |
| | Acidi carbol. cryst., - - | 0.1 |
| | Eucain. hydrochlor. B., - - | 0.7 |
| | Cocain. hydrochlor., - - | 0.3 |
| | Aquæ destill., - - | ad 100.fi |

M.

The solution can be sterilized and kept for a long time without deteriorating. The technique of injection is the same as for Schleich's cocaine infiltration anesthesia. The special value claimed for this solution is the absence of a secondary vasomotor dilatation following the primary vasomotor contraction produced by the cocaine. Gelatine acts as the hamostatic.—*Dental Review*.

